# OpenIMC: an open-source platform for analyzing single-cell and spatial proteomics by imaging mass cytometry

**DOI:** 10.21203/rs.3.rs-9558630/v1

**Published:** 2026-05-12

**Authors:** Dean Tessone, Mohamed Kamal, Valerie Hennes, James Hicks, Peter Kuhn

**Affiliations:** 1 Convergent Science Institute in Cancer, University of Southern California, Los Angeles, CA, 90089, USA; 2 Department of Biological Sciences, University of Southern California, Los Angeles, CA, 90089, USA; 3 USC Norris Comprehensive Cancer Center, Keck School of Medicine, University of Southern California, Los Angeles, CA, 90089, USA; 4 Department of Aerospace and Mechanical Engineering, University of Southern California, Los Angeles, CA, 90089, USA; 5 Department of Biomedical Engineering, University of Southern California, Los Angeles, CA, 90089, USA; 6 Department of Urology, Keck School of Medicine, University of Southern California, Los Angeles, CA, 90089, USA

**Keywords:** Imaging Mass Cytometry, Single-cell proteomics, Spatial proteomics, Image analysis, Multiplexed imaging, Open-source software, Batch correction

## Abstract

Imaging Mass Cytometry (IMC) enables spatially resolved single-cell proteomics, but fragmented data analysis tools limit reproducibility. We present OpenIMC, an open-source platform consolidating IMC workflows into a cohesive environment. OpenIMC integrates visualization, preprocessing, segmentation, feature extraction, phenotyping, and spatial analysis. Accessible via graphical and command-line interfaces sharing a backend, the platform ensures consistent execution. Built for scalability and reproducibility, OpenIMC automatically records analytical parameters and enables sharing of complete sessions. Case studies analyzing blood-derived cells and tissue slices validate the software platform by recovering known phenotypes and structural organization. OpenIMC lowers technical barriers, supporting rigorous, scalable single-cell and spatial proteomics.

## Background

Spatially resolved single-cell proteomics enables quantitative characterization of tissue organization and molecular heterogeneity^1^. Imaging Mass Cytometry (IMC) is a prominent technology in this space, enabling simultaneous measurement of forty protein markers at subcellular resolution without spectral overlap^2^. By coupling laser ablation with time-of-flight mass spectrometry, IMC generates multiplexed images that preserve molecular and spatial context across thousands of cells^2,3^. This capability has facilitated studies of cellular interactions in cancer, immunity, and development^4,5,6^.

Despite its analytical potential, IMC data analysis remains a bottleneck for broader adoption and reproducible use^7,8^. Ion-count intensities follow Poisson-like noise distributions and are affected by shot noise^2,9^, hot pixels^10^, and isotopic channel spillover^11^. These properties motivate careful normalization for quantitative comparison^8,10^. Segmentation is further complicated by faint or discontinuous cellular boundaries^12^, motivating both classical and deep-learning approaches^12–17^.

A diverse computational ecosystem currently supports IMC analysis. Recent work by Windhager et al. improved data processing by integrating community-developed methods into a reproducible programmatic workflow^13^. Similarly, modular systems such as MCMICRO offer scalable pipelines^18^. Despite these advances, comprehensive IMC analysis still requires coordinating multiple independent software packages for visual inspection^10,13,19^, quality control^20,21^, spillover correction^11^, denoising^9,22^, segmentation^12–17^, feature extraction^13,23^, clustering^24^, and spatial analysis^21,25^. Relying on custom scripts and intermediate file formats across disparate environments limits accessibility and complicates reproducibility^7^. These steps necessitate interactive visual inspection, rapid iteration, and consistent data handling, capabilities that limit accessibility and complicate reproducibility within disjointed analytical environments.

OpenIMC is an open-source framework built on interoperable community standards^13,25^ that resolves this fragmentation. It integrates the complete analytical pipeline, encompassing assay development, preprocessing, quality control, segmentation, feature extraction, phenotyping, spatial analysis, and publication-ready analysis and visualization within a unified and reproducible environment.

## Implementation

**Figure 1.**
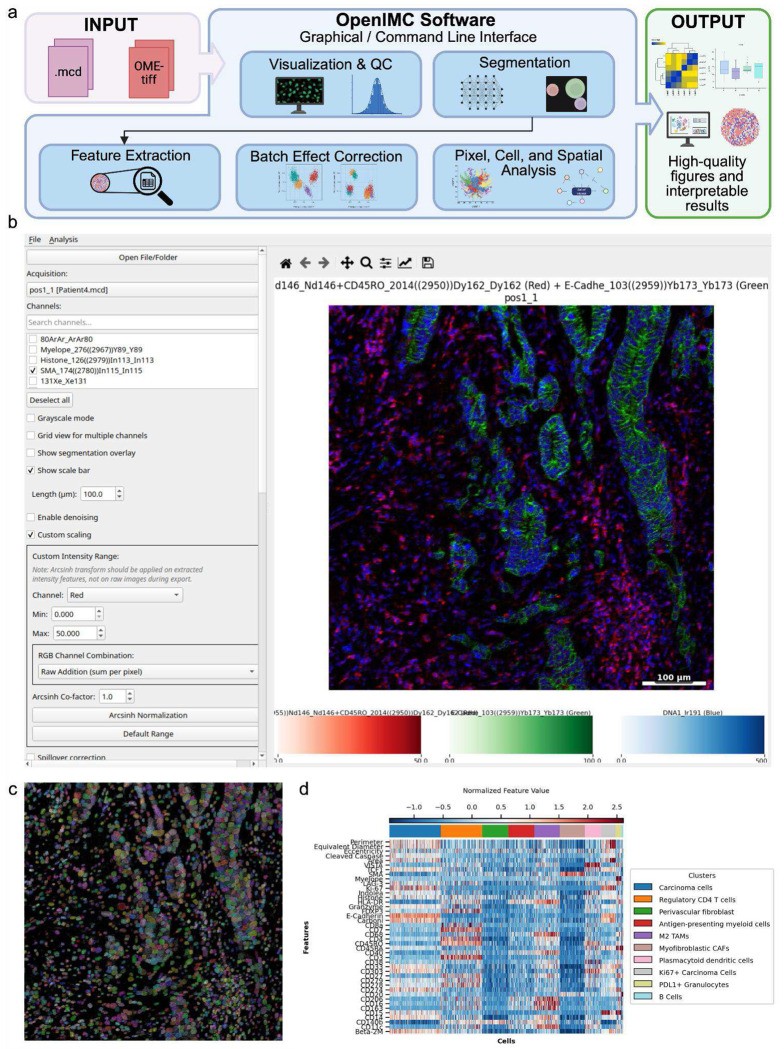
Overview of the OpenIMC platform for IMC analysis. A. End-to-end workflow implemented in OpenIMC, from data import and preprocessing to segmentation, feature extraction, batch correction, cell clustering and phenotyping, and spatial analysis. B. Screenshot of the GUI image viewer, channel selection, and preprocessing controls, with an RGB image of a tissue slice displayed (CD45RO and CD45RA in red, E-Cadherin in green, and DNA in blue). C. Segmentation overlay on top of the DNA channel, where segmentation is performed using DeepCell’s CellSAM model. D. Representative analysis output of a single cell feature heatmap. The workflow in A was created with BioRender.com and B, C, and D were created in OpenIMC and assembled in BioRender.com.

OpenIMC is an integrated analysis platform designed to support the full lifecycle of an Imaging Mass Cytometry (IMC) experiment, from assay development and image inspection through single-cell and spatial analysis, within a unified and reproducible framework (Figure 1A). OpenIMC is developed in python version 3.11, with the front-end graphical user interface implemented with PyQt5. OpenIMC is distributed under the GPLv3 license and is publicly available at https://github.com/dean-tessone/OpenIMC. Detailed software dependencies and versioning are provided in the repository.

The platform natively supports standard IMC data formats, including proprietary .mcd files (leveraging readimc^13^), OME-TIFF exports^26,27^, spillover matrices^11^, and externally generated segmentation masks. Multiple samples can be analyzed concurrently, with metadata consistently linked to images, segmentation masks, and derived single-cell features.

For assay development and data inspection, OpenIMC provides an interactive viewer for visualization of individual channels, composites, and simultaneous comparisons of regions of interest (Figure 1B). Quantitative quality-control metrics, including signal-to-noise estimates, enable early identification of underperforming channels^13^. Preprocessing workflows include hot-pixel removal, background filtering, isotopic spillover correction, and optional variance stabilization^9,13,19,22^. Users select from multiple segmentation backends spanning classical, machine-learning, and deep-learning approaches^14,15,17^, with parameters and the channels to mask on adjustable within a single session for iterative refinement (Figure 1C).

Following segmentation, OpenIMC supports feature extraction (quantifying both morphological and marker features), batch correction, clustering, and phenotyping using methods including k-means, HDBSCAN, Louvain, and Leiden clustering (Figure 1D)^28,29,30^. Spatial analyses operate on the same single-cell representations and include neighborhood enrichment, distance-based metrics, and spatial clustering approaches, leveraging existing spatial analysis libraries such as Squidpy^4,21,25^.

OpenIMC additionally provides native support for high-resolution IMC (HR-IMC), a recently introduced acquisition strategy that achieves subcellular resolution through repeated laser ablation of the same tissue region^3,31^. Additional experimental optimization is required prior to analysis of biological samples for HR-IMC, where users optimize laser energies to generate a deconvolution map from control data that characterizes signal attenuation across successive ablation passes. This deconvolution map is then applied to experimental data to reconstruct high-resolution images. The platform quantifies signal attenuation across successive laser passes and fits a sigmoidal decay model to control data to guide laser energy selection (Supplementary Figure S1). Application of the Richardson-Lucy deconvolution kernel can also be done from within OpenIMC, circumventing another external workflow.

## Results

### Reproducibility and computational performance

OpenIMC produces deterministic outputs across graphical and command-line interfaces through a shared computational backend. Under controlled conditions with fixed parameters and software versions, segmentation workflows using Cellpose^15^ and CellSAM^17^ yielded identical outputs across repeated runs, with perfect concordance across all evaluated metrics (Supplementary Table 1, Part 1). We further evaluated consistency between interfaces by comparing results generated through the graphical user interface and command-line interface using identical inputs and parameters. Segmentation masks and downstream feature extraction outputs were indistinguishable between interfaces (Supplementary Table 1, Part 2) and extracted single-cell features showed perfect correspondence (Supplementary Table 1, Part 3).

To support reproducibility in practice, OpenIMC implements automated provenance tracking, recording analytical parameters, software versions, and execution settings for each session. Complete analysis states, including raw data, segmentation masks, and derived features, are exportable and reloadable to enable full reconstruction of analytical workflows.

Computational performance scales with dataset size^32,33^ and available hardware resources. Parallelization of CPU-bound tasks significantly improves throughput for segmentation preprocessing and feature extraction, while maintaining stable memory usage (Supplementary Figure S2; Supplementary Figure S3). Segmentation accuracy and feature extraction outputs are consistent with established pipelines (i.e. Steinbock^13^, Supplementary Figures S3 and S4). The platform extracts upwards of 300 features per single cell at significantly faster run times than existing workflows (Supplementary Figure S4).

### Comparison to existing tools

A wide range of software tools support portions of the IMC workflow, but most operate on isolated steps and require users to assemble complex multi-tool pipelines before reaching interpretable results. A recent review of IMC analysis tools identified key analytical steps provided by various software: image visualization, cell segmentation, data pre-processing/batch correction, feature extraction, cell phenotyping, spatial analyses, and pixel-level analysis^7^. OpenIMC provides an integrated analysis platform that provides key steps necessary for complete analysis and improves on many existing features (Table 1). A comprehensive description of our methodology for comparing to existing tools can be found in Methods.

Vendor software (MCDViewer) enables inspection of raw .mcd files but does not provide quantitative analysis. Visualization tools such as napari-IMC^13^ allow interactive exploration but depend heavily on custom Python code or plugins for segmentation, feature extraction, and downstream analysis. histoCAT^10^ offers exploratory clustering and simple neighborhood analysis but relies on precomputed segmentation masks and does not support modern batch correction or multi-slide harmonization.

Preprocessing and segmentation are addressed by tools such as readIMC, the IMC Segmentation Pipeline, and steinbock, which implement workflows using Ilastik^14^, CellProfiler^23^, and deep-learning models^13,15,16^. These approaches provide robust segmentation and feature extraction but typically require separate environments for visualization, quality control, and downstream analysis. Workflow systems such as MCMICRO^18^ enable scalable processing in HPC or containerized settings but are less suited for interactive use.

For downstream analysis, imcRtools provides IMC-specific preprocessing and integration within the Bioconductor ecosystem^13^, while Squidpy enables advanced spatial analyses on precomputed feature matrices^25^. These tools offer strong functionality but do not provide a unified end-to-end environment, whereas OpenIMC enables users to move directly from raw .mcd files to final analyses within a single interactive session.

OpenIMC integrates data access and analysis within a single framework while remaining interoperable with community tools, preserving compatibility with established methods such as existing IMC-specific packages and Squidpy. This reduces the need for manual data transfer and custom scripting across environments while preserving compatibility with established methods.

### Application: Proteomic Profiling of Circulating Cells

To demonstrate the application of OpenIMC in a single-cell proteomic setting, we applied the platform to an IMC dataset generated from nucleated cells collected from peripheral blood liquid biopsies from four patients with late-stage breast cancer. Prior work has used IMC to interrogate circulating tumor cell biology in similar liquid biopsy contexts^34–39^. Briefly, nucleated cells were plated as monolayers, stained with a four-channel immunofluorescence (IF) panel consisting of DAPI, Pan-Cytokeratin, Vimentin, and CD45/CD31^35^, and regions containing candidate rare cells were identified using an anomaly detection algorithm^40^ prior to IMC acquisition. For the purposes of this study, we generated IMC analysis of cells negative for pan-cytokeratin (non-CTCs) in IF. All subsequent image processing, segmentation, feature extraction, quality control, and downstream analyses were performed entirely within OpenIMC.

**Figure 2.**
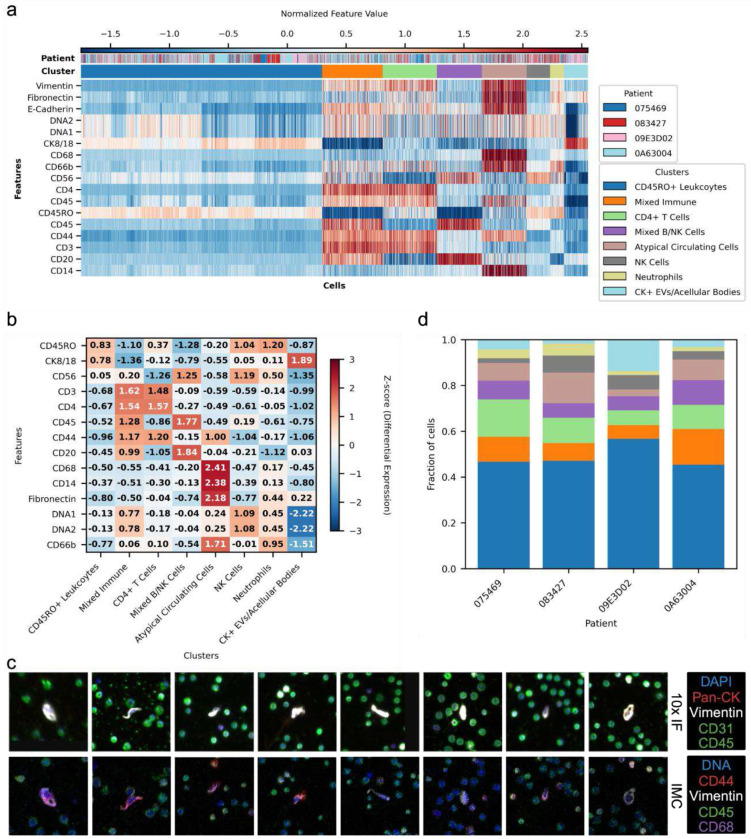
Single-cell analysis within OpenIMC reveals a coherent circulating atypical population. A. Unsupervised Leiden clustering (resolution=0.3) of z-scored, arcsinh-transformed mean intensities and morphometric single-cell features, resolving a distinct Cytokeratin-negative, Vimentin-high population across all patients. B. Alternative visualization of z-score highlighting enrichment of Fibronectin, elevated CD68, and comparatively low CD45RO and CD45RA in the identified population. Black boxes are the 3 highest (or more than 3 with ties) differential markers for a cluster. C. Paired immunofluorescence (IF) and IMC crops for rare events identified by the IF rarity detector, demonstrating correspondence between Vimentin-positive, Cytokeratin-negative IF events and their IMC counterparts. Cells form a cohesive morphological type in IF, indicating a biologically consistent, easily discoverable cell type that we have expanded on utilizing multiplexed proteomics. IF data is captured with a 10x objective on a scanning microscope and is displayed as a color composite with DAPI in blue, pan-Cytokeratin (Pan-CK) in red, Vimentin in white, CD45 in green, and CD31 in green (CD45 and CD31 are multiplexed in the same IF channel). IMC data is displayed as a composite image with DNA in blue, CD44 in red, Vimentin in white, CD45 in green, and CD68 in magenta. D. Per-patient abundance of all cellular population, showing recurrence of the atypical population across all four individuals. All visualization, segmentation, feature extraction, batch correction, and downstream analysis (A, B, D) were performed entirely within OpenIMC.

Unsupervised clustering of single-cell IMC intensities identified a distinct population of Vimentin-high cells across all four patients (Figure 2A). These cells exhibited a hybrid phenotype characterized by the co-expression of multiple lineage markers. Differential protein intensity analysis and single-cell visualization confirmed the enrichment of Fibronectin within and immediately surrounding these cells (Supplementary Figure S5). Furthermore, this population displayed elevated expression of E-cadherin, CD68, CD66b, CD44, and CD14, while maintaining low levels of the leukocyte markers CD45RO and CD45RA (Figure 2A, 2B). This specific combination of epithelial (E-cadherin), mesenchymal (Vimentin), and myeloid (CD68) markers is highly non-canonical in peripheral blood. We designate these cells as atypical circulating cells (ACCs).

To relate the upstream IF-based rarity detection to the IMC-derived phenotypes, we assembled paired IF–IMC image crops for the same candidate events. Despite the limited marker content of the IF panel, paired visualization demonstrated that oblong, Vimentin-positive, Cytokeratin-negative cells identified by the rarity detector aligned with the corresponding atypical cluster in IMC feature space, with heterogeneity in CD68 and CD44 expression (Figure 2C). Notably, this population does not represent doublets or clear segmentation faults. This population was observed across all four patients (Figure 2D).

This case study demonstrates OpenIMC’s capacity to support the end-to-end interrogation of rare circulating cells by bridging IF-based rarity detection with high-dimensional proteomic profiling. By consolidating the entire analytical lifecycle, from quality control and segmentation to batch correction, into a unified environment, OpenIMC enables the reproducible characterization of rare populations from liquid biopsies. Specifically, the platform addressed the challenges of segmenting cells with highly heterogeneous marker expression and disparate morphological scales. Furthermore, OpenIMC’s scalable architecture permitted the integration of multiple patient cohorts into a single interactive analysis, a workflow that previously required extensive custom scripting and manual data harmonization.

### Application: Tissue-based spatial analysis

**Figure 3.**
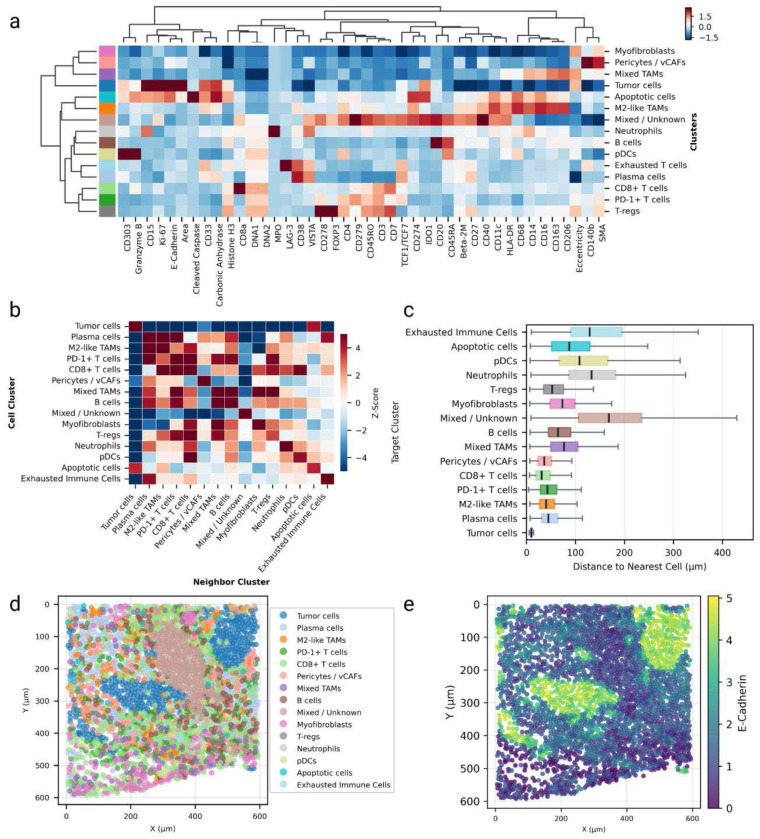
OpenIMC facilitates spatial analysis of the breast tumor microenvironment. A. Clustermap of Leiden clustered single cells from a breast tissue sample, yielding 15 cellular phenotypes. Colorbar is z-scored mean protein intensities and morphometric features. B. Neighborhood enrichment analysis using a k-nearest neighbor graph (k=20) and 999 permutations. The heatmap displays z-scores of observed versus expected neighborhood frequencies, highlighting a compartmentalized tissue architecture and significant avoidance between malignant and stromal subsets. C. Euclidean distance distributions from tumor cells to the nearest neighboring cell of each target phenotype. Boxplots represent the median, interquartile range (IQR), and 1.5 × IQR (whiskers). D. Clusters visualized spatially on a scatter plot of a tissue slice. E. Mean, single-cell intensity of E-Cadherin visualized on a scatter plot of the same tissue slice as in D. All spatial statistics and visualizations were performed within the OpenIMC graphical interface.

Beyond single-cell phenotyping, a central utility of IMC lies in its capacity to quantify the architectural logic of the tumor microenvironment (TME). However, the technical barriers to performing statistically rigorous spatial modeling often necessitate extensive computational expertise. These challenges range from high-density segmentation to graph-based neighborhood analysis. OpenIMC democratizes these complex workflows by providing an automated and end-to-end framework for spatial interrogation. To demonstrate the spatial modules of OpenIMC, we analyzed a breast cancer specimen from the IMMUcan study^13,32,41^. Unsupervised clustering resolved 15 cellular phenotypes, including malignant epithelial cells, myofibroblasts, and a diverse immune landscape (Figure 3A).

To quantify the structural organization of the TME, we utilized the graph-based modules in OpenIMC. Spatial analysis using a k-nearest neighbor graph (k=20) revealed that tumor cells (E-Cadherin+, Carbonic Anhydrase+, Ki-67+, Figure 3A) form highly cohesive, compartmentalized niches spatially distinct from the surrounding stroma (Figure 3B). This compartmentalization was statistically validated using Moran’s I to measure spatial autocorrelation. Epithelial markers exhibited the highest degree of spatial structure, with E-cadherin (Moran’s I≈0.70) showing significant local clustering (FDR-corrected p<0.001; Supplementary Figure S6). These results confirm the presence of organized protein domains and demonstrate OpenIMC’s ability to perform statistically rigorous spatial modeling.

Neighborhood enrichment analysis further resolved the organizational patterns of the TME. Tumor cells exhibited significant self-enrichment and co-occurrence with apoptotic tumor cells, while maintaining global avoidance of most stromal populations (Figure 3B). However, per-cell distance distributions revealed a stratified peri-tumoral architecture. While the median distance to neutrophils exceeded 100 μm, CD8+ and PD-1+ T cells occupied a significantly more proximal niche, typically within 35-50 μm of the tumor boundary (Figure 3C). This nuanced proximity suggests a model of restricted immune infiltration where specific populations are recruited to the tumor periphery but remain excluded from the malignant core. vCAFs/pericytes are also present in the same spatial proximity, where they may play a role in vascularization and matrix remodeling adjacent to the tumor boundary.

Consistent with this excluded phenotype, co-occurrence probability curves demonstrated a sharp decay in homotypic association for malignant cells (Supplementary Figure S7). This trajectory indicates that tumor cells are restricted to cohesive nests with a characteristic spatial scale of approximately 50-100 μm. In contrast, the co-occurrence scores for immune subsets relative to the malignant population remained consistently low across all measured radii, providing quantitative evidence of a spatially restricted and immune-excluded TME. OpenIMC also enables visualization of spatial organization of both individual markers and clusters, demonstrating clear organization of malignant epithelial cells into excluded, distinct niches within the breast tissue (Figure 3D, 3E). By integrating these spatial metrics and visualizations into a unified workflow, OpenIMC enables the systematic characterization of the spatial logic governing the tumor-immune interface without the need for custom coding.

## Discussion

OpenIMC provides a cohesive analytical environment for Imaging Mass Cytometry. A critical liability in existing IMC literature is the prevalence of unlogged parameters, including normalization constants and filtering thresholds. OpenIMC aligns spatial proteomic research with FAIR principles^42^ by implementing automated provenance tracking as a core feature. This ensures that complex spatial findings are directly linked to explicit computational states rather than obscured by ad hoc manual interventions.

A central contribution of OpenIMC is the consolidation of the entire IMC analytical lifecycle within a single interoperable system. This integration allows steps that previously required the coordination of disparate software packages to be handled within a unified framework. Furthermore, OpenIMC extends analysis capabilities both upstream and downstream of conventional pipelines. By incorporating tools for antibody titration and signal-to-noise evaluation, the platform addresses critical quality control needs that are often overlooked in standard workflows. These features are particularly relevant as the field transitions toward higher-resolution acquisition with the newly published HR-IMC protocol. In this context, signal restoration and deconvolution become essential for accurate subcellular localization and the recovery of biological signals that would otherwise be lost to blurring. OpenIMC has already integrated and established a robust pipeline for HR-IMC analysis, including upstream signal attenuation calculations and downstream deconvolution.

Our benchmarking results demonstrate that OpenIMC maintains a high degree of concordance with established tools while offering superior scalability. The parallelization of CPU-bound tasks improves throughput for segmentation and feature extraction without introducing stochastic variability. This performance indicates that OpenIMC offers a practical balance for large-scale studies that require both computational efficiency and rigorous validation against existing community standards. The modular architecture, implemented via abstract base classes, ensures that OpenIMC remains an extensible integration layer. This structure allows the platform to evolve alongside methodological advances, such as the emergence of foundation models for segmentation, rather than functioning as a closed or static reimplementation.

Beyond typical CTCs and WBCs, the OpenIMC pipeline identified atypical circulating cells (ACCs) in breast cancer patients characterized by a non-canonical CD45+/CD14+/CD68+/CD66b+/E-cadherin+/vimentin+ profile. This phenotype suggests a transitional biological state co-expressing myeloid, epithelial, and mesenchymal markers, potentially arising from tumor-immune interactions or cell fusion events. While other circulating hybrid populations like cancer-associated macrophage-like cells have been documented, the precise nature of ACCs remains unclear. Future genomic and single-cell transcriptomic analyses are necessary to determine clonal concordance with the primary tumor and define the specific role of these cells in the metastatic cascade.

Several limitations should be noted. Although OpenIMC integrates multiple established segmentation backends, no currently available segmentation model is explicitly optimized for the ion-count distributions, noise characteristics, and acquisition physics unique to IMC. Existing methods such as Cellpose^15^ and CellSAM^17^ perform well in practice but are primarily trained on optical microscopy data. The development of IMC-specific segmentation architectures represents an important direction for future work. Furthermore, as IMC resolution increases, standard morphometric features may require replacement by learned morphological embeddings to fully capture subcellular architecture^43^. Additionally, it is important to note that our spatial analysis vignette presented in this study serves as a methodological validation on a single specimen, however the framework is architected to scale across the extensive cohorts typical of modern clinical trials. Finally, while GUI and CLI platforms cannot entirely replace the flexibility of raw code, OpenIMC provides a reproducible foundation for standard acquisition routines and scientists seeking a more accessible computational entry point.

In summary, OpenIMC replaces fragmented and script-heavy pipelines with a cohesive and extensible framework. By bridging the gap between interactive data exploration and scalable command-line execution, OpenIMC standardizes the interrogation of high-dimensional spatial proteomics. As the field moves toward higher-resolution imaging and larger clinical cohorts, such unified environments will be essential for translating complex spatial data into reproducible biological insights.

## Availability and Requirements

Project name: OpenIMC

Project home page: https://github.com/dean-tessone/OpenIMC

Operating system(s): Cross-platform (Linux, macOS, Windows)

Programming language: Python

Other requirements: Python >=3.11, dependencies listed in repository (requirements.txt)

License: GPLv3

Any restrictions to use by non-academics: None

## Methods

### Use of Generative AI

Generative AI was used to assist in writing aspects of the OpenIMC application source code. Code generation was conducted using the Cursor IDE with a mixture of AI models (Claude Opus 4.5 and 4.6, OpenAI GPT 4 and Codex 5.3, and Cursor Composer 1, 1.5, and 2). Generative AI was used to edit this manuscript for conciseness and clarity.

### Documentation

Complete documentation describing the code, architecture, and analysis modules of OpenIMC is available at https://dean-tessone.github.io/OpenIMC/overview.html. Readers can find details describing the implementation and calculations behind visualization modules, pre-processing, signal-to-noise ratio calculations, segmentation, feature extraction, and all downstream analyses. We provide a detailed tutorial video walking through the core functionality and a sample analysis at https://youtu.be/CKSwJE3jdi0.

### Reproducibility Analysis

To maximize reproducibility, OpenIMC was implemented around shared core functions that are used by both the graphic interface and the command-line interface (exposed through openimc.core). We assessed the outputs of the graphical interface and command-line interface for both segmentation and feature extraction on a dataset of four patients provided by Windhager et al. as a tutorial dataset for IMC processing. For segmentation, the two DNA channels (DNA1 and DNA2) were passed through either CellSAM or Cellpose cyto 3. Default parameters were used for both CellSAM and Cellpose cyto 3. Three replicates were used for each ROI. On a per-ROI basis, Dice coefficient, Aggregated Jaccard Index (AJI), object count, object area, boundary displacement, and Hausdorff distance were computed. We evaluated both the reproducibility of each method (Cellpose and CellSAM) on the same interface (command line) and across interfaces (graphical vs command-line) for each metric.

Dice coefficient is the spatial overlap between two binary masks X and Y, ranging from 0 to 1 where 1 indicates complete spatial overlap between the two masks. Dice $(X,Y)=\frac{2|X\cap Y|}{\left|X|+|Y\right|}$. The Aggregated Jaccard Index (AJI) is an instance-level similarity metric computed as the sum of intersections between reference objects and their best-matched predicted counterparts, divided by the sum of their unions plus the area of all unmatched predicted pixels. $\mathrm{AJI}=\frac{\sum_{i=1}^{L}\left|G_{i}\cap s_{{*}_{j}}(i)\right|}{\sum_{i=1}^{L}\left|G_{i}\cup S{*}_{j}(i)\right|+\sum_{k\in K}\left|S_{k}\right|}$ where $G_{i}$ is the ith object in the reference set $G,S{*}_{j}(i)$ is the ith object in the predicted set S that has the maximum overlap with $G_{i},K$ is the set of predicted objects that have no match in the reference set, L is the total number of objects in the reference set. Hausdorff distance measures the maximum distance mismatch between two sets of points, identifying the point on the boundary of one mask that is farthest from any point on the boundary of the other, where smaller values indicate better boundary alignment. $d_{H}\left(B_{X},B_{Y}\right)=\max\left\{\sup_{b_{x}\in B_{X}}\inf_{b_{y}\in B_{Y}}d\left(b_{x},b_{y}\right),\sup_{b_{y}\in B_{Y}}\inf_{b_{x}\in B_{X}}d\left(b_{x},b_{y}\right)\right.$ where $B_{X}$ and $B_{Y}$ are the sets of pixels on the boundaries of the two mask sets X and Y respectively, $d\left(b_{x},b_{y}\right)$ is the Euclidean distance between two boundary points, sup (supremum) and inf (infimum) represent the maximum and minimum distances. Boundary displacement is a measure of the average boundary agreement. $BD\left(B_{X},B_{Y}\right)=\frac{1}{\left|B_{X}\right|+\left|B_{Y}\right|}\left(\sum_{b_{x}\in B_{X}}d\left(b_{x},B_{Y}\right)+\sum_{b_{y}\in B_{Y}}d\left(b_{y},B_{X}\right)\right)$, where d(b,B) is the shortest distance from a point b to any point on the boundary B and |B| is the number of pixels on the respective boundary.

### Performance Benchmarking

To benchmark the performance of OpenIMC’s segmentation and feature extraction workflows, we utilized the command line arguments, wrapped with basic functions to change the number of workers that were involved in multiprocessing. Performance metrics were collected using Python’s time module for wall-clock time measurement and psutil (≥ 5.9.0) for monitoring peak RAM usage and maximum resident set size (RSS). For segmentation benchmarks, GPU memory usage was tracked using PyTorch’s CUDA memory management when GPU acceleration was available. We measured the end-to-end wall-time and the peak RAM to either segment cells or extract features as a function of either the number of images processed (50, 100, 200, 500) or the number of workers (1, 4, 8, 16, 22). For comparative benchmarking of feature extraction, we also evaluated steinbock (version 0.19.0), a command-line tool for IMC data analysis, using the ‘/usr/bin/time -v’ utility to measure wall-clock time and memory consumption for the ‘steinbock measure intensities’ and ‘steinbock measure regionprops’ commands. All benchmarks were run with multiple repeats (n=3) to ensure reproducibility.

### Accuracy Benchmarking

For the purposes of this work, segmentation deep learning models were not fine-tuned or used for transfer-learning for ion-count images. We assess the provided models, as they are, for accuracy. We benchmark the masks generated by CellSAM to those generated in the original Jackson et al. publication^33^ (via ilastik) through the Dice Coefficient, Aggregated Jaccard Index (AJI), and object count correlations. We note that neither represents a ground truth (i.e. pathologist annotations) but rather we benchmark model accuracy against published data.

For feature extraction, we benchmark against the readily established steinbock^13^ pipeline, noting the feature-level concordance for both morphology and intensity features. We compute mean absolute error (MAE), a line of best fit (R^2^), and spearman correlation.

### Comparison to Existing Tools

We performed a systematic benchmark of ten established computational frameworks utilized for Imaging Mass Cytometry (IMC) data analysis: MCDViewer, napari-IMC^13^, histoCAT^10^, readimc^13^, IMC Segmentation Pipeline^13^, steinbock^13^, CellProfiler^23^, MCMICRO^18^, imcRtools^13^, and Squidpy^25^. Support for seven core analytical tasks was classified as full (+), partial (±), or none (−) based on the native execution of algorithms within the software namespace. Full support required that a framework satisfy all task-specific prerequisites natively. Partial support was assigned if a framework satisfied at least one but not all prerequisites, or if it required a non-native plugin. Frameworks satisfying zero prerequisites were classified as having no support. Software were primarily characterized based on their documentation, where possible. Features not explicitly documented were tested on PCs running Ubuntu 24.04, MacOS Tahoe 26.1, or Windows 11.

The criteria for each analytical task were rigorously defined to distinguish between core functionality and peripheral compatibility. Image visualization required the native capacity to parse both proprietary .mcd files and standardized OME-TIFF exports, render single-channel grayscale data, and assemble multi-channel composite images. Cell segmentation support was defined by the native execution of mathematical or deep learning algorithms, such as watershed, StarDist, Ilastik, or Cellpose, to generate masks. Feature extraction necessitated the automated generation of quantified data matrices containing both morphometrics and integrated marker intensities per segmented cell.

Downstream analytical capabilities were evaluated based on their statistical rigor and biological relevance. Batch effect correction required the native integration of established harmonization algorithms, such as ComBat^44^ or Harmony^45^, to explicitly regress out technical covariates. Standard scalar transformations, including mean centering or z-scoring, were insufficient for this classification. Single-cell analysis support required the native execution of unsupervised clustering or sequential marker gating to map phenotypic assignments to individual cells. Spatial analysis was defined as the mathematical computation of cellular neighborhoods and the statistical evaluation of pairwise spatial interactions between cell types. Finally, pixel analysis required the calculation of spatial cross-correlation matrices for marker pairs evaluated at pixel resolution, independent of cellular segmentation boundaries. For a broader review of the IMC software landscape and general data analysis strategies, readers are directed to Milosevic et al.^7^

### Circulating Rare Cell Analysis

Eight mL of peripheral blood was collected from four patients with late-stage breast cancer in 10 mL tubes (Streck, Cell-free DNA BCT) at the clinical site, shipped to USC, and processed according to previously described protocols. In brief, isotonic ammonium chloride (A649-3, Fisher Scientific) was used to lyse the erythrocyte population and the cellular fraction was plated on glass slides (Marienfeld, Lauda, Germany) at a density of approximately 2.5 million nucleated cells / slide, yielding between 8-16 slides per patient. One slide from each patient was subsequently stained with a four-color immunofluorescence assay consisting of 4’,6 diamidino–2-phenylindole (DAPI, D1306, Thermo Fisher), a pan-Cytokeratin antibody cocktail (C2562-.5ML, Sigma Aldrich and M088801-2, Dako North American, conjugated to AlexaFluor^®^ 555), Vimentin (9854BC, Cell Signaling Technology, conjugated to AlexaFluor^®^ 488), CD45 (MCA87A647, AbD Serotec, conjugated to AlexaFluor^®^; 647), and CD31 (clone: WM59, MCA1738A647, BioRad, conjugated to AlexaFluor^®^ 647). Slides were stained using the IntelliPATH FLX autostainer. After staining, slides were imaged on custom scanning microscopes with 10x objectives.

To identify candidate rare cells on each peripheral blood sample, we employed a deep-learning based rare event detection algorithm^40^. In brief, slides are cartesianally tiled, passed through a denoising autoencoder, and the reconstruction error of the autoencoder is measured as a proxy for rarity. This approach avoids segmentation, thereby enabling the robust detection of cells of various morphometric phenotypes in the blood. We collected the top 2,500 rare tiles on each slide, manually curated them to remove imaging artifacts, and subsequently selected a sample to profile with IMC. We note the denoising autoencoder and other analysis related to immunofluorescence was used to select the cells for further IMC analysis, and no subsequent analysis relies on immunofluorescence imaging.

IMC was conducted on a CyTOF Hyperion imaging mass cytometer (Standard Biotools). All antibody clones used in this experiment are included in Supplementary Table 2. All antibodies which could not be acquired pre-conjugated to metals were conjugated in the lab using a Maxpar antibody labeling kit.

Raw .mcd files from each slide were analyzed within OpenIMC’s graphical interface by leveraging the capability to analyze all slides in one session. Cells were segmented using CellSAM with ‘gauge cell size’ enabled. The DNA1 and DNA2 channels were used for nuclei and the CD45, CD45RO, CD45RA, Vimentin, and CK8/18 channels were used for cytoplasm/membrane. Channels were denoised with hot-pixel removal and combined by normalizing each with min-max normalization followed by a simple mean as an input to CellSAM. Subsequently, OpenIMC’s quality control module was used to assess the quality of each marker. Cytoplasmic or nuclear markers with signal to noise ratio under 5 were excluded from analysis (Supplementary Figure S8). Secreted factors (Fibronectin) were maintained for analysis. Visual inspection was additionally used to assess antibodies that bound unspecifically and these were excluded from further analysis (SMA).

All channels were denoised using OpenIMC’s hot-pixel removal module prior to feature extraction. Intensity and morphometric features were extracted using OpenIMC’s feature extraction module (computes statistics of intensity and morphometrics from each masked region in an image) and arcsinh scaled with a cofactor of 1.0. Batch effect correction was performed with Harmony^45^ within OpenIMC, with each slide acting as a batch. Harmony was performed on a PCA space of the original data with 90% of the variance maintained. In brief, Harmony clusters the PCA space and assesses that there is sufficient representation of each co-variate/batch within each cluster and subsequently optimizes a correction to move cells in PCA space to achieve sufficient representation, with a maximum of 20 iterations or until convergence (converged in 9 iterations for this dataset).

Each cell was clustered based on the Z-scored mean intensity of the channels using the Leiden algorithm on a K-NN graph with 15 neighbors (resolution = 0.3). Clusters with similar features primarily driven by minor differences in intensity within the same marker positive channels or small differences in area were merged manually. Clustering results prior to manual merging are presented in Supplementary Figure S9. Visualizations of the different cluster identities were generated using OpenIMC’s heatmaps, dimensionality reduction, differential expression, and enumeration visualizations. Cell types were manually annotated by analysis of the marker and morphology profiles and comparison to known literature on circulating cells in the blood of breast cancer patients. The complete analytical provenance, as exported by OpenIMC, is available within the deposited data repository.

### Breast cancer spatial analysis

A breast cancer tissue sample from the IMMUcan study was analyzed using OpenIMC^13,32,41^. All analysis was performed within OpenIMC’s graphical interface. Masks were generated using CellSAM. Both DNA channels (DNA1, DNA2) and Histone H3 were used in conjunction as the nuclear channel. CD45RA, CD163, CD20, CD68, CD3, CD45RO, CD8a, Carbonic Anhydrase, CD4, CD14, and E-Cadherin were used in conjunction for the cytoplasm/membrane. For all channel combinations, each channel was normalized using min-max normalization, denoised to remove hot pixels, and then combined with a simple mean. CellSAM was run with default bounding box threshold (0.4) and with the ‘Gauge cell size’ parameter turned on. After segmentation, features were extracted and arcsinh normalized with a coefficient of 1.0.

For single-cell phenotyping, we implement a widely used approach: outliers are clipped at the 99th percentile and the remaining features are z-scored for clustering and analysis. Cells touching the edge of an acquisition were excluded from analysis. We implement a generic version of the PhenoGraph^24^ algorithm building a jaccard-weighted euclidean k-NN graph of 15 neighbors and applying the Leiden community detection algorithm on the graph. After excluding edge events, a total of 9,542 cells remained from the patient sample. The Leiden algorithm identified 19 clusters. Clusters were subsequently annotated into a given cell type based on marker expression and morphological features by manual inspection. Clusters annotated into the same phenotype were manually merged (3 tumor cell clusters merged, 2 plasma cell clusters merged, 2 CD8+ T cell clusters merged) to yield 15 total clusters.

Spatial analysis is implemented through OpenIMC functions and through Squidpy. For Squidpy based analyses, ROI-specific AnnData objects were generated from single-cell centroid coordinates. Centroid coordinates are converted to micrometers from pixels (pixel size = 1 micrometers in this analysis). A spatial graph was constructed using a k-NN based approach, with k=20. Neighborhood enrichment was computed per ROI with a permutation test (n=1000) on the spatial connectivity matrix, comparing observed cluster-cluster adjacency with label-shuffled null distributions. Spatial autocorrelation was assessed with a global Moran’s I test (n=999 permutations). Distance distribution analysis was performed per ROI by computing, for each cell, the nearest neighbor distance in each cluster, excluding itself for a within-cluster comparison using a KD-tree based search. Co-occurrence was computed with Squidpy across increasing distance thresholds (20-200 micrometers). Co-occurrence statistic is the probability ratio p(exp|cond)/p(exp), where values greater than 1 indicate over-representation of a target phenotype at a given distance, values less than 1 indicate under-representation or avoidance, and around 1 indicate values near the expectation. Expectation (p(exp)) is the global probability of the given cell type, excluding any distance calculation. Moran’s I was used to compute the local auto-correlation of each channel by evaluating the degree to which a feature’s value at a given location correlates with the values of its k nearest neighbors (k=20), where a positive coefficient indicates spatial clustering of similar values and a negative coefficient indicates a dispersed, chess-board-like distribution relative to a null hypothesis of complete spatial randomness.

## Supplementary Material

This is a list of supplementary files associated with this preprint. Click to download.

• SupplementaryFigures.docx

## Figures and Tables

**Table 1.** Comparison of Major IMC Software tools across visualization, segmentation, feature extraction, batch effect correction, single-cell analysis, spatial analysis, and pixel-level correlation analysis.

Tool	Viz.	Seg.	Feat.	Batch	SC	Spatial	Pixel	OS
**OpenIMC**	**+**	**+**	**+**	**+**	**+**	**+**	**+**	**CP**
MCDViewer	+	−	−	−	−	−	−	Windows
napari-IMC	+	±	±	−	−	−	−	CP
histoCAT	+	+	+	−	+	+	−	Mac, Windows
readimc	±	−	−	−	−	−	−	CP
IMC Seg. Pipeline	−	+	+	−	−	−	−	CP
steinbock	−	+	+	−	−	−	−	CP
CellProfiler	±	+	+	−	−	−	+	CP
MCMICRO	±	+	+	−	+	−	−	CP
imcRTools with cytomapper	±	−	−	−	±	+	−	CP
Squidpy	±	+	+	±	±	+	−	CP

Viz. = visualization, Seg. = segmentation, Feat. = feature extraction, Batch = batch effect correction, SC = single-cell analysis, Spatial = spatial analysis, Pixel = pixel-level correlation analysis, OS = operating system. + = full support, ± = partial support, − = no support. CP = cross platform. Wrapping around an existing algorithm or tool for any task is still characterized as full support. Squidpy receives partial support on certain tasks due to its integration with the broader Scanpy ecosystem.

## Data Availability

All raw imaging mass cytometry (IMC) data, segmentation masks, and downstream analytical files generated in this study are deposited in Zenodo (DOI: 10.5281/zenodo.19713713.). This repository also includes masks and feature tables generated for reproducibility and accuracy benchmarking. Access to external reference datasets is provided through their respective original sources: IMMUcan images were obtained from Windhager et al. (https://doi.org/10.5281/zenodo.7575859)^13,32^, and comparative spatial data and masks were acquired from Jackson et al. (https://doi.org/10.5281/zenodo.3518284)^4,33^. Sample high-resolution IMC data is provided by Whipman et al. (https://zenodo.org/records/17077712)^31^. The source code for OpenIMC is available at https://github.com/dean-tessone/OpenIMC. A tagged release of OpenIMC consistent with the version of the software at the time of writing is available on github as a tagged release: https://github.com/dean-tessone/OpenIMC/releases/tag/1.0.0 and deposited in Zenodo (DOI: https://doi.org/10.5281/zenodo.19713161).

## List of Abbreviations

IMC: Imaging Mass Cytometry
HR-IMC: High resolution imaging mass cytometry
ACC: atypical circulating cells
AJI: aggregated Jaccard index
CLI: Command-line interface
CTC: circulating tumor cell
FDR: false discovery rate
GUI: graphical user interface
IF: Immunofluorescence
IQR: interquartile range
k-NN: k-nearest neighbors
MCD: mass cytometry data format
OME: open microscopy environment
QC: quality control
ROI: region of interest
SNR: signal-to-noise ratio
TME: tumor microenvironment
WBC: white blood cell

## References

[1] Method of the Year 2024: spatial proteomics. Nat Methods 21, 2195–2196 (2024). 10.1038/s41592-024-02565-3 39643689

[2] Giesen C., Wang H., Schapiro D. Highly multiplexed imaging of tumor tissues with subcellular resolution by mass cytometry. Nat Methods 11, 417–422 (2014). 10.1038/nmeth.2869 24584193

[3] Bollhagen A., Whipman J., Coelho R. High-resolution imaging mass cytometry to map subcellular structures. Nat Methods 22, 2601–2608 (2025). 10.1038/s41592-025-02889-8 41168486 PMC12695663

[4] Jackson H.W., Fischer J.R., Zanotelli V.R.T. The single-cell pathology landscape of breast cancer. Nature 578, 615–620 (2020). 10.1038/s41586-019-1876-x 31959985

[5] Damond N., Engler S., Zanotelli V.R.T., Schapiro D., Wasserfall C.H., Kusmartseva I., Nick H.S., Thorel F., Herrera P.L., Atkinson M.A. & Bodenmiller B. A map of human type 1 diabetes progression by imaging mass cytometry. Cell Metabolism 29, 755–768.e5 (2019). 10.1016/j.cmet.2018.11.014 30713109 PMC6821395

[6] Li N., van Unen V., Guo N., Abdelaal T., Somarakis A., Eggermont J., Mahfouz A., Chuva de Sousa Lopes S.M., Lelieveldt B.P.F. & Koning F. Early-life compartmentalization of immune cells in human fetal tissues revealed by high-dimensional mass cytometry. Frontiers in Immunology 10, 1932 (2019). 10.3389/fimmu.2019.01932 31474997 PMC6703141

[7] Milosevic V. Different approaches to imaging mass cytometry data analysis. Bioinformatics Advances 3, vbad046 (2023). 10.1093/bioadv/vbad046 (academic.oup.com) 37092034 PMC10115470

[8] Hunter B., Nicorescu I., Foster E., McDonald D., Hulme G., Fuller A., Thomson A., Goldsborough T., Hilkens C.M.U., Majo J., Milross L., Fisher A., Bankhead P., Wills J., Rees P., Filby A. & Merces G. OPTIMAL: an optimized imaging mass cytometry analysis framework for benchmarking segmentation and data exploration. Cytometry A 105, 36–53 (2024). 10.1002/cyto.a.24803 37750225 PMC10952805

[9] Lu P., Oetjen K.A., Bender D.E. IMC-Denoise: a content aware denoising pipeline to enhance Imaging Mass Cytometry. Nat Commun 14, 1601 (2023). 10.1038/s41467-023-37123-6 36959190 PMC10036333

[10] Schapiro D., Jackson H., Raghuraman S. histoCAT: analysis of cell phenotypes and interactions in multiplex image cytometry data. Nat Methods 14, 873–876 (2017). 10.1038/nmeth.4391 28783155 PMC5617107

[11] Chevrier S., Crowell H.L., Zanotelli V.R.T., Engler S., Robinson M.D. & Bodenmiller B. Compensation of signal spillover in suspension and imaging mass cytometry. Cell Systems 6, 612–620.e5 (2018). 10.1016/j.cels.2018.02.010 29605184 PMC5981006

[12] Xiao X., Qiao Y., Jiao Y., Fu N., Yang W., Wang L., Yu R. & Han J. Dice-XMBD: deep learning-based cell segmentation for imaging mass cytometry. Frontiers in Genetics 12, 721229 (2021). 10.3389/fgene.2021.721229 34603385 PMC8480472

[13] Windhager J., Zanotelli V.R.T., Schulz D. An end-to-end workflow for multiplexed image processing and analysis. Nat Protoc 18, 3565–3613 (2023). 10.1038/s41596-023-00881-0 37816904

[14] Berg S., Kutra D., Kroeger T. ilastik: interactive machine learning for (bio)image analysis. Nat Methods 16, 1226–1232 (2019). 10.1038/s41592-019-0582-9 31570887

[15] Stringer C., Wang T., Michaelos M. Cellpose: a generalist algorithm for cellular segmentation. Nat Methods 18, 100–106 (2021). 10.1038/s41592-020-01018-x 33318659

[16] Greenwald N.F., Miller G., Moen E. Whole-cell segmentation of tissue images with human-level performance using large-scale data annotation and deep learning. Nat Biotechnol 40, 555–565 (2022). 10.1038/s41587-021-01094-0 34795433 PMC9010346

[17] Marks M., Israel U., Dilip R. CellSAM: a foundation model for cell segmentation. Nat Methods 22, 2585–2593 (2025). 10.1038/s41592-025-02879-w 41360960 PMC12695629

[18] Schapiro D., Sokolov A., Yapp C. MCMICRO: a scalable, modular image-processing pipeline for multiplexed tissue imaging. Nature Methods 19, 311–315 (2022). 10.1038/s41592-021-01308-y 34824477 PMC8916956

[19] Ijsselsteijn M.E., Somarakis A., Lelieveldt B.P.F., Höllt T. & de Miranda N.F.C.C. Semi-automated background removal limits data loss and normalizes imaging mass cytometry data. Cytometry A 99, 1187–1197 (2021). 10.1002/cyto.a.24480 34196108 PMC9542015

[20] Eling N., Damond N., Hoch T. & Bodenmiller B. cytomapper: an R/Bioconductor package for visualization of highly multiplexed imaging data. Bioinformatics 36, 5706–5708 (2020). 10.1093/bioinformatics/btaa1061 PMC8023672 33367748

[21] Rombaut B., Defauw A., Vernaillen F., Mortier J., Van Hamme E., Van Gassen S., Seurinck R. & Saeys Y. Scalable analysis of whole slide spatial proteomics with Harpy. Bioinformatics, btag122 (2026). 10.1093/bioinformatics/btag122 PMC13064853 41826800

[22] Baranski A., Milo I., Greenbaum S., Oliveria J.P., Mrdjen D., Angelo M. & Keren L. MAUI (MBI analysis user interface): an image processing pipeline for multiplexed mass based imaging. PLoS Computational Biology 17, e1008887 (2021). 10.1371/journal.pcbi.1008887 33872301 PMC8084329

[23] Stirling D.R., Swain-Bowden M.J., Lucas A.M., Carpenter A.E., Cimini B.A. & Goodman A. CellProfiler 4: improvements in speed, utility and usability. BMC Bioinformatics 22, 433 (2021). 10.1186/s12859-021-04344-9 34507520 PMC8431850

[24] Levine J.H., Simonds E.F., Bendall S.C., Davis K.L., Amir el-AD, Tadmor M.D., Litvin O., Fienberg H.G., Jager A., Zunder E.R., Finck R., Gedman A.L., Radtke I., Downing J.R., Pe’er D. & Nolan G.P. Data-driven phenotypic dissection of AML reveals progenitor-like cells that correlate with prognosis. Cell 162, 184–197 (2015). 10.1016/j.cell.2015.05.047 26095251 PMC4508757

[25] Palla G., Spitzer H., Klein M. Squidpy: a scalable framework for spatial omics analysis. Nat Methods 19, 171–178 (2022). 10.1038/s41592-021-01358-2 35102346 PMC8828470

[26] Linkert M., Rueden C.T., Allan C., Burel J.M., Moore W., Patterson A., Loranger B., Moore J., Neves C., Macdonald D., Tarkowska A., Sticco C., Hill E., Rossner M., Eliceiri K.W. & Swedlow J.R. Metadata matters: access to image data in the real world. Journal of Cell Biology 189, 777–782 (2010). 10.1083/jcb.201004104 20513764 PMC2878938

[27] Goldberg I.G., Allan C., Burel J.M., Creager D., Falconi A., Hochheiser H., Johnston J., Mellen J., Sorger P.K. & Swedlow J.R. The Open Microscopy Environment (OME) data model and XML file: open tools for informatics and quantitative analysis in biological imaging. Genome Biology 6, R47 (2005). 10.1186/gb-2005-6-5-r47 15892875 PMC1175959

[28] McInnes L., Healy J. & Astels S. hdbscan: hierarchical density based clustering. Journal of Open Source Software 2, 205 (2017). 10.21105/joss.00205

[29] Traag V.A., Waltman L. & van Eck N.J. From Louvain to Leiden: guaranteeing well-connected communities. Sci Rep 9, 5233 (2019). 10.1038/s41598-019-41695-z 30914743 PMC6435756

[30] Blondel V.D., Guillaume J.-L., Lambiotte R. & Lefebvre E. Fast unfolding of communities in large networks. Journal of Statistical Mechanics: Theory and Experiment 2008, P10008 (2008). 10.1088/1742-5468/2008/10/P10008

[31] Whipman J, Bollhagen A. Repository for high-resolution imaging mass cytometry data. Zenodo; 2025. 10.1038/s41592-025-02889-8 PMC12695663 41168486

[32] Eling N, Windhager J. Example imaging mass cytometry raw data. Zenodo; 2023.

[33] Jackson Hartland W., Fischer Jana R., Zanotelli Vito R.T, Ali H. Raza, Mechera Robert, Soysal Savas D., The Single-Cell Pathology Landscape of Breast Cancer. Zenodo; 2019. 10.1038/s41586-019-1876-x 31959985

[34] Gerdtsson E., Pore M., Thiele J.A., Gerdtsson A.S., Malihi P.D., Nevarez R., Kolatkar A., Velasco C.R., Wix S., Singh M., Carlsson A., Zurita A.J., Logothetis C., Merchant A.A., Hicks J. & Kuhn P. Multiplex protein detection on circulating tumor cells from liquid biopsies using imaging mass cytometry. Convergent Science Physical Oncology 4, 015002 (2018). 10.1088/2057-1739/aaa013 30906572 PMC6430142

[35] Chai S., Matsumoto N., Storgard R., Peng C.C., Aparicio A., Ormseth B., Rappard K., Cunningham K., Kolatkar A., Nevarez R., Tu K.H., Hsu C.J., Malihi P., Corn P., Zurita A., Hicks J., Kuhn P. & Ruiz-Velasco C. Platelet-coated circulating tumor cells are a predictive biomarker in patients with metastatic castrate-resistant prostate cancer. Molecular Cancer Research 19, 2036–2045 (2021). 10.1158/1541-7786.MCR-21-0383 34462330 PMC12747416

[36] Higa N., Limb A., Hennes V. Simultaneous expression of epithelial and immune cell markers in circulating tumor cells identified in patients with stage 4 breast cancer.Commun Med 5, 309 (2025). 10.1038/s43856-025-01024-0 40707771 PMC12290075

[37] Naghdloo A., Kamal M., Tessone D., Hennes V., Hicks J. & Kuhn P. Caught in the act: tumor–immune interactions in circulation of patients with immune marker positive circulating tumor cells. Cancers 17, 3667 (2025). 10.3390/cancers17223667 41301033 PMC12651602

[38] Pore M., Balamurugan K., Atkinson A., Breen D., Mallory P., Cardamone A., McKennett L., Newkirk C., Sharan S., Bocik W. & Sterneck E. Assessment of imaging mass cytometry (IMC) as a tool to characterize circulating tumor cells (CTCs) in preclinical mouse models. bioRxiv (2025). 10.64898/2025.12.18.695262

[39] Naghdloo A., Tessone D., Nagaraju R.M. Representation learning enables robust single cell phenotyping in whole slide liquid biopsy imaging. Sci Rep 15, 36589 (2025). 10.1038/s41598-025-20514-8 41120558 PMC12540859

[40] Murgoitio-Esandi J., Tessone D., Naghdloo A. Unsupervised detection of rare events in liquid biopsy assays. npj Precis. Onc. 9, 225 (2025). 10.1038/s41698-025-01015-3 PMC12228807 40617936

[41] Eling N., Dorier J., Rusakiewicz S., Liechti R., Devanand P., Daniel M., Windhager J., Fernandez B.P., Déglise S., Despland L., Benyagoub A., Możejko M., Uchal D., Szczurek E., Loboda A., Sandkuijl D., Parsotam N., Hong H.S., Morfouace M., Guex N., Coukos G., Bodenmiller B., Tissot S. & Schulz D. Multi-modal image analysis for large-scale cancer tissue studies within IMMUcan. Cell Reports Methods 5, 101170 (2025). 10.1016/j.crmeth.2025.101170 40930089 PMC12539258

[42] Wilkinson M., Dumontier M., Aalbersberg I. The FAIR Guiding Principles for scientific data management and stewardship. Sci Data 3, 160018 (2016). 10.1038/sdata.2016.18 26978244 PMC4792175

[43] Wenckstern J., Jain E., Cheng Y., von Querfurth B., Vasilev K., Pariset M., Cheng P.F., Liakopoulos P., Michielin O., Wicki A., Gut G. & Bunne C. AI-powered virtual tissues from spatial proteomics for clinical diagnostics and biomedical discovery. arXiv (2025). 10.48550/arXiv.2501.06039

[44] Zhang Y., Parmigiani G. & Johnson W.E. ComBat-seq: batch effect adjustment for RNA-seq count data. NAR Genomics and Bioinformatics 2, lqaa078 (2020). 10.1093/nargab/lqaa078 33015620 PMC7518324

[45] Korsunsky I., Millard N., Fan J. Fast, sensitive and accurate integration of single-cell data with Harmony. Nat Methods 16, 1289–1296 (2019). 10.1038/s41592-019-0619-0 31740819 PMC6884693

